# The burden of multiple sclerosis: A community health survey

**DOI:** 10.1186/1477-7525-6-1

**Published:** 2008-01-07

**Authors:** C Allyson Jones, Sheri L Pohar, Sharon Warren, Karen VL Turpin, Kenneth G Warren

**Affiliations:** 1 Faculty of Rehabilitation Medicine, University of Alberta, Edmonton, Alberta, T6G 2G4, Canada; 2 Institute of Health Economics, Edmonton, Alberta, T5J 3N4, Canada; 3 Faculty of Medicine and Dentistry, University of Alberta, Edmonton, Alberta, T6G 2G3, Canada

## Abstract

**Background:**

Health-related quality of life (HRQL) in persons with multiple sclerosis (MS) who reside within the community relative to the general population is largely unknown. Data from the Canadian Community Health Survey Cycle 1.1 (CCHS 1.1) were used to compare HRQL of persons with MS and the general population.

**Methods:**

A representative sample of adults (18 years or older) from the cross sectional population health survey, CCHS 1.1, was examined to compare scores on the Health Utilities Index Mark 3 (HUI3), a generic preference-based HRQL measure, of respondents with (n = 302) and without (n = 109,741) MS. Selected sociodemographic covariates were adjusted for in ANCOVA models. Normalized sampling weights and bootstrap variance estimates were used in the analysis.

**Results:**

The mean difference in overall HUI3 scores between respondents with and without MS was 0.25 (95% CI: 0.20, 0.31); eight times greater than the clinically important difference. The largest differences in scores were seen with the ambulation (0.26; 95% CI: 0.20, 0.32) and pain attributes (0.14; 95% CI: 0.09, 0.19). Clinically important differences with dexterity and cognition were also observed.

**Conclusion:**

While the proportion of the Canadian population with MS is relatively small in comparison to other diseases, the magnitude of the burden is severe relative to the general population.

## Background

The diverse symptoms associated with multiple sclerosis (MS) adversely impact health-related quality of life (HRQL) which, in turn, is manifested in extensive physical, psychosocial and economic burden [1-3]. Although the assessment of HRQL in MS is well recognized as an important clinical assessment tool [4], burden of morbidity of persons with MS in comparison with the general population is largely unknown.

The Expanded Disability Status Scale (EDSS) is the primary disease specific health measure for MS [5], but it is heavily weighted toward ambulation and is unable to provide a broader comparison of HRQL attributes among different conditions and the general population. The use of a generic health measure to complement the disease specific health measure is typically advocated for the appraisal of the overall impact of MS.

The evaluation of HRQL of persons with MS has been primarily in clinical or patient study groups. Relying solely on these cohorts limits the external validity of these findings and generates possible selection bias [6]. Few investigations have compared the burden of illness in MS to a reference group or to the general population [7-10]. Subsequently, only some studies have made comparisons by statistically adjusting for differences between persons with MS and a reference population. Limited evidence indicates that physical attributes such as ambulation are lower in persons with MS than the general population; however, it is unclear whether other attributes such as pain and emotion are relatively lower than the general population. Using the SF-36, lower scores were reported for only physical dimensions in persons with MS as compared to the US population; however, mental health scores were comparable to the general population [8]. Alternatively, both physical and mental health components were lower for patients with MS than the Norwegian general population [7]. Others have reported problems with balance, cognition, visual disturbance, bowel and bladder difficulties, spasticity, depression, anxiety, bipolar disorders, speech problems and fatigue for persons with MS who reside within the community [9,11,12].

The comparison of HRQL in the general population to a sample of persons with MS provides quantitative baseline estimates of the impact of MS which, in turn, can be used for therapeutic intervention, program and healthcare evaluations. The primary aim of this study was to compare the HRQL of persons who have MS to those persons without MS, using a cross-sectional representative sample of the general population. A secondary aim was to identify the attributes associated with the burden of MS relative to the general population adjusting for various socio-demographic factors. To assist in identifying the effect of MS independent of other comorbidities, we also compared the HRQL of persons with MS alone to the health status of respondents without any chronic medical conditions.

## Methods

### Survey source

Data from the Canadian Community Health Survey Cycle 1.1 (CCHS 1.1) were used in this analysis. The CCHS 1.1 is a cross-sectional survey that collected data pertaining to utilization of health services, determinants of health and health status from 2000 to 2001 in the Canadian population over age 12 [13]. All information collected in the CCHS 1.1 was either self-reported or reported by a proxy respondent. The survey excludes individuals living on crown or reserve land, in institutions, members of the Canadian Armed Forces and some remote areas of the country, but still represents approximately 98% of the Canadian population over 12 years of age [13].

A multistage stratified cluster design combined with random sampling methods was used to select a representative sample of the Canadian population [13]. Interviews could be completed either in person or by telephone [14]. At the end of Cycle 1.1 a total of 131, 535 respondents had been surveyed; the overall response rate was 84.7% [14]. Approval to access the survey data was obtained from Statistics Canada and ethical approval was obtained through the University of Alberta Health Research Ethics Board.

### Sample

In the CCHS 1.1 respondents were asked to identify chronic medical conditions that were diagnosed by a healthcare professional and were or were expected to be present for at least 6 months. A variety of chronic conditions including MS were listed. Of the 131, 535 respondents surveyed, 335 respondents reported having a diagnosis of MS consistent with this definition. This proportion represented a weighted percentage of 0.22% of the community dwelling Canadian population over age 12 which is comparable to prevalence rates reported [12,15]. The analysis was restricted to adult respondents (18 years or older) who had complete data (n = 109,741 respondents without MS and n = 302 respondents with MS).

### Measures

The Health Utilities Index Mark 3 (HUI3), a generic preference-based measure was used to evaluate HRQL [16-19]. The concept of the HUI is based on functional capacity rather than performance. The HUI3 health states are defined by eight attributes (vision, hearing, speech, ambulation, dexterity, emotion, cognition and pain and discomfort), with 5 or 6 levels of functioning for each attribute. Single attribute utility scores for each of the eight attributes range from 0.0 to 1.0, with a score of 0.0 representing the lowest level of functioning on an attribute and a score of 1.0 representing full functional capacity on an attribute. A difference of 0.05 on a single attribute is considered to be clinically important [18]. For descriptive purposes, classification systems are established for aggregating attribute levels into none/mild (0.89 to 1.00), moderate (0.70 to 0.88) and severe (less than 0.70) [18]. The morbidity burden of a single attribute can also be depicted by the distribution of persons at each level.

An overall score for health states of the HUI3 is also generated which range from -0.36 to 1.0 (-0.36 = worst possible health, 0.0 = dead and 1.0 = perfect health). A difference of 0.03 on overall HUI3 scores is considered to be clinically important [18]. In order to assign meaning to an average overall HUI3 scores, the scores can be grouped into categories reflecting level of impairment: none/mild (0.89 to 1.00), moderate (0.70 to 0.88) and severe (less than 0.70) [20].

The HUI3 has been used in both clinical and population health studies [18]. It was originally developed for use in the 1990 Ontario Health Survey conducted by Statistics Canada and has subsequently been used for national population health surveys [18]. While other generic health measures have reported floor and ceiling effects in MS cohorts, the HUI3 has been reported to be robust in this patient population [1,6,21].

### Analysis

Descriptive statistics were used to summarize the characteristics of the two groups, that is, respondents who reported MS and those who did not. The sample sizes reported are the actual observed numbers; however, the reported percentages are weighted by the sampling weights provided by Statistic Canada. The χ^2 ^value was used for statistical comparison of proportions between the two groups. Overall HUI3 scores and single attribute scores of respondents with and without MS were then compared using analysis of covariance (ANCOVA), with adjustment for age, sex, education, marital status, social assistance as a source of income, and number chronic medical conditions other than MS [22-24]. As the proportion of respondents who failed to report total household income was large and would reduce the cohort, social assistance was used as a marker of income.

To better capture the disease burden associated with MS alone, a subgroup analysis was also performed, where the health status of respondents with MS only (n = 60) and respondents without MS or any other self-reported chronic medical conditions was compared (n = 33,975 respondents). Differences in overall and single attribute utility scores for these subgroups were also assessed using ANCOVA, with adjustment for age, sex, education, marital status, and social assistance as a source of income.

Sampling weights were applied to all analyses in order to account for the unequal probability of being selected into the survey [14]. Bootstrap variance estimates were used to adjust for clustering and stratification [14] and to estimate 95% confidence intervals and p-values. Bootstrapping is a technique to estimate the variance, that is, to approximate the sampling distributions of the statistic. Repeated random samples are drawn with replacement from the observations to obtain a set of estimates. All analyses were performed using WESTVAR version 4.2, with bootstrap weights provided by Statistics Canada.

## Results

In this community-based population, respondents with MS were approximately four years older on average than the general population (48.7 versus 44.8 years of age, p < 0.05) (Table 1). A larger proportion of respondents with MS than respondents without MS was female (68.3% versus 50.9%, p < 0.05). Unadjusted overall HUI3 scores were considerably lower for respondents with MS (0.57 versus 0.88); this difference was more than 10 times that would be considered clinically important (Table 1). Clinically important differences were also seen with the single attributes; MS respondents had lowers scores for ambulation, dexterity, cognition and pain (Table 1).

**Table 1:** Demographic characteristics

	MS	General Population
Age – mean (95% CI)	48.7 (46.6 to 50.8)*	44.8 (44.7 to 44.8)
Sex (% Female)	68.3*	50.9
Education – %		
Less than high school	17.9	22.2
High School	19.0	20.4
Some post-secondary/college/trade school	42.9	36.5
University degree	20.1	20.9
Marital Status (% Married)	71.0	64.3
Social Assistance (% Receiving Social Assistance)	11.1*	5.1
Number of other Medical Conditions mean (95% CI)	2.6 (2.3 to 3.0)*	1.6 (1.6 to 1.6)
HUI3 Scores – mean (95% CI)†		
Overall HUI3 score	0.57 (0.52 to 0.63)*	0.88 (0.88 to 0.88)
Vision	0.93 (0.91 to 0.95)*	0.97 (0.97 to 0.97)
Hearing	0.99 (0.98 to 0.99)	0.99 (0.99 to 0.99)
Speech	0.99 (0.99 to 0.99)	1.00 (0.99 to 1.00)
Ambulation	0.71 (0.65 to 0.77)*	0.98 (0.98 to 0.98)
Dexterity	0.93 (0.90 to 0.96)*	1.00 (1.00 to 1.00)
Emotion	0.93 (0.91 to 0.95)*	0.97 (0.97 to 0.97)
Cognition	0.89 (0.86 to 0.92)*	0.96 (0.96 to 0.96)
Pain	0.75 (0.69 to 0.80)*	0.93 (0.93 to 0.93)

* p < 0.05

† HUI3 classification systems for overall and attribute levels: none/mild (0.89 to 1.00), moderate (0.70 to 0.88) and severe (less than 0.70).

After adjusting for the model covariates (age, sex, education, marital status, and social assistance), clinically important differences in overall HUI3 scores and single attribute scores persisted. The mean difference in overall HUI3 scores between respondents with and without MS was reduced from 0.31 to 0.25 after adjustments (95% confidence interval (CI): 0.20 to 0.31, p < 0.05) (Table 2). This difference, however, was still more than eight times what would be considered clinically important. On the single attributes, a difference of 0.05 is considered clinically meaningful. Particularly large differences in scores were observed for ambulation and pain, with differences of 0.26 (95% CI: 0.20 to 0.32, p < 0.05) and 0.14 (95% CI: 0.09 to 0.19, p < 0.05), being observed, respectively. Clinically important differences on the dexterity and cognition attributes were also observed, although differences were not as large as those observed for ambulation and pain (Table 2). No clinical differences were seen with sensory and emotion attributes.

**Table 2:** Adjusted† mean scores and differences in overall and single attribute utility scores for respondents with and without MS

	MS	General Population	Mean Difference (95% CI)
Overall HUI3 score	0.58	0.84	0.25 (0.20 – 0.31)*
Vision	0.94	0.96	0.03 (0.01 – 0.05)*
Hearing	0.99	0.99	0.00 (-0.01 – 0.004)
Speech	0.99	1.00	0.01 (-0.003 – 0.006)
Ambulation	0.72	0.98	0.26 (0.20 – 0.32)*
Dexterity	0.93	1.00	0.06 (0.03 – 0.10)*
Emotion	0.92	0.95	0.03 (0.01 – 0.05)*
Cognition	0.89	0.94	0.05 (0.02 – 0.09)*
Pain	0.77	0.91	0.14 (0.09 – 0.19)*

† Adjusted for age, sex, education, marital status, social assistance, and number of medical conditions other than MS

* p < 0.05 based on the Bootstrap Variance Estimate for between groups difference after adjusting for covariates.

When persons with MS alone were compared to persons without MS or any other chronic conditions, differences in overall and single attribute utility scores were similar to those observed in the entire sample (Table 3). The difference in overall HUI3 scores between the subgroups without any chronic conditions was 0.29 (95% CI: 0.18 to 0.41, p < 0.05) (Table 3). Again, the largest differences on the single attributes were observed for ambulation and pain (Table 3). Clinically important differences were also observed on the dexterity and cognition attributes.

**Table 3:** Adjusted† mean scores and differences in overall and single attribute utility scores for respondents with and without MS, but no other chronic conditions

	MS	General Population	Mean Difference (95% CI)
Overall HUI3 score	0.64	0.93	0.29 (0.18 to 0.41)*
Vision	0.95	0.98	0.03 (-0.01 to 0.07)*
Hearing	1.00	0.99	0.00 (0.00 to -0.01)
Speech	0.99	1.00	0.00 (-0.01 to 0.02)
Ambulation	0.69	1.00	0.31 (0.17 to 0.44)*
Dexterity	0.95	1.00	0.05 (-0.01 to 0.11)
Emotion	0.93	0.97	0.04 (-0.01 to 0.09)*
Cognition	0.88	0.97	0.09 (0.02 to 0.16)*
Pain	0.88	1.00	0.11 (0.03 to 0.20)*

† Adjusted for age, sex, education, marital status, and social assistance

* p < 0.05 based on the Bootstrap Variance Estimate for between groups difference after adjusting for covariates.

## Discussion

Within the context of a national population health survey, the burden of illness for persons with MS was quantified using a generic health measure, HUI3 in the community dwelling population. We found that the MS population experienced large deficits in overall HRQL relative to the general population without MS. When the effect of other chronic conditions was removed, persistent large deficits of HRQL existed for persons with MS. Given the underlying neuropathologic changes that occur to the central nervous system and the diverse clinical features, it is not surprising that HRQL would be affected by the disease. In particular, our findings quantify significant difficulties with pain, ambulation, dexterity, and cognition in persons with MS.

Our findings were similar to other population samples. A Norwegian community-based cohort reported lower health status in persons with MS compared to the general population [7]. Findings from a cross sectional survey also reported lower physical functioning, vitality, general health and psychological domains in MS patients than controls [25,26]. An association between MS and mental health has been also reported within other population-based samples [12,27]. Although SF-36 physical component scores in persons with MS were lower in comparison to the US population, the mental component scores were similar to the general population [8]. This divergence from other studies may be attributable, in part, to psychometric properties of the components scores for the SF-36. Orthogonal factor rotation is used in the determination of the SF-36 component scores, that is, mental and physical components scores are treated as independent. Subsequently, the algorithm has been shown to significantly under-estimate mental health of patients with MS as compared to the component scores based on the RAND-36 Health Status Inventory [28].

While the prevalence of pain in MS is well recognized, the severity relative to the general population has been examined by few investigators [29]. We reported large differences in pain for both unadjusted and adjusted analysis. The difference between the MS and general population while adjusting for other covariates was almost five times that would be considered clinically important. Others have recognized acute and chronic pain in this patient population as a substantial clinical problem [29-31], while others have not [7,8]. Congruent with our findings, Svendsen and colleagues reported that the severity of pain is greater in persons with MS than the general population [29]. The ramifications of pain are also far reaching given the associations with depression, fatigue, and poorer health status [30,32]. The assessment and treatment of pain warrants further consideration in this patient population which may directly improve HRQL.

The HRQL of persons with MS reported in this study illustrates that the HRQL is worse than HRQL reported with many other diseases. Maddigan and colleagues reported the overall HUI3 score for persons with diabetes, heart disease, arthritis or stroke ranged from 0.74 to 0.89 [22]. Moreover, the overall HUI3 scores of various combinations using three of these four conditions still ranged from 0.62 to 0.66 [22]. In relative terms, one may conclude that the HRQL of burden associated with MS is substantially higher than any one of these four other chronic conditions or in any combination of three of these four conditions. Others have also reported that patients with MS are among one of the most severely impaired in comparison with other chronic conditions such as cardiovascular conditions, cancer, endocrinologic conditions, and chronic respiratory diseases [6,33]. This illustrates the severe impairment that is associated with MS, even among community dwelling individuals with the disease.

Although secondary analysis makes use of valuable data, the limitations of these findings are noteworthy. First, ascertainment of MS was via self-report. Although questions regarding the presence of medical conditions specified that the condition was diagnosed by a health professional, there remained potential for individuals to over- or under-report any medical condition, including MS. Likewise, no disease specific health measure for MS or indicator for disease course was included in the CCHS 1.1.

Another limitation of this study concerned the number of respondents who were missing data on covariates and were excluded from the analysis. While this was less than 10.0% of MS respondents, generalizability of these results to the respondents with missing data may be limited. Despite over 98% of the Canadian community dwelling population being represented in the survey, the generalizability of the results to the entire Canadian population with MS is limited by the fact that the sampling frame would not capture those individuals who reside in institutions or on reserve lands. That being said, the true HRQL burden of the entire Canadian population with MS would be under-estimated by these results given that individuals with MS who resided in institutions were more likely to have greater impairment than those residing in the community. Although the impact of MS appears to be more severe in First Nations People, the prevalence rates of MS are relatively low [34] and would likely have a small impact on the overall HRQL of this sample population.

## Conclusion

These findings highlight the severity of impairment expressed by persons with MS relative to the general population and when compared to other chronic conditions. While the proportion of persons with MS may be relatively small in relation to the Canadian population, the issue of HRQL in MS patients is important from clinical practice and policy decision perspectives. These findings, which identify the diverse impairment and quantify the amount of disability persons with MS, can be used when evaluating therapeutic interventions and healthcare programs.

## Abbreviations

HRQL: Health-related quality of life; 

MS: multiple sclerosis; 

CCHS 1.1: Canadian Community Health Survey Cycle 1.1; 

HUI3: Health Utilities Index Mark 3; 

ANCOVA: analysis of covariance; 

95%CI: 95% confidence interval; 

EDSS: Expanded Disability Status Scale.

## Competing interests

The author(s) declare that they have no competing interests.

## Authors' contributions

Drs Jones and Pohar were responsible for the conception of the study. Dr. Pohar analyzed the data. Dr. Jones drafted the article. All authors contributed to the interpretation of the results and revising the article for important intellectual content. All authors read and approved the final manuscript.

## References

[B1] Mitchell AJ, Benito-Leon J, Gonzalez JM, Rivera-Navarro J (2005). Quality of life and its assessment in multiple sclerosis: integrating physical and psychological components of wellbeing. Lancet Neurol.

[B2] Patwardhan MB, Matchar DB, Samsa GP, McCrory DC, Williams RG, Li TT (2005). Cost of multiple sclerosis by level of disability: a review of literature. Mult Scler.

[B3] (1998). Burden of illness of multiple sclerosis: Part I: Cost of illness. The Canadian Burden of Illness Study Group. Can J Neurol Sci.

[B4] Vickrey BG, Hays RD, Genovese BJ, Myers LW, Ellison GW (1997). Comparison of a generic to disease-targeted health-related quality-of-life measures for multiple sclerosis. J Clin Epidemiol.

[B5] Kurtzke JF (1983). Rating neurologic impairment in multiple sclerosis: an expanded disability status scale (EDSS). Neurology.

[B6] (1998). Burden of illness of multiple sclerosis: Part II: Quality of life. The Canadian Burden of Illness Study Group. Can J Neurol Sci.

[B7] Nortvedt MW, Riise T, Myhr KM, Nyland HI (1999). Quality of life in multiple sclerosis: measuring the disease effects more broadly. Neurology.

[B8] Pittock SJ, Mayr WT, McClelland RL, Jorgensen NW, Weigand SD, Noseworthy JH, Rodriguez M (2004). Quality of life is favorable for most patients with multiple sclerosis: a population-based cohort study. Arch Neurol.

[B9] Ford HL, Gerry E, Johnson MH, Tennant A (2001). Health status and quality of life of people with multiple sclerosis. Disabil Rehabil.

[B10] Minden SL, Frankel D, Hadden LS, Srinath KP, Perloff JN (2004). Disability in elderly people with multiple sclerosis: An analysis of baseline data from the Sonya Slifka Longitudinal Multiple Sclerosis Study. NeuroRehabilitation.

[B11] Roessler RT, Rumrill PD, Hennessey ML, Vierstra C, Pugsley E, Pittman A (2003). Perceived strengths and weaknesses in employment policies and services among people with multiple sclerosis: results of a national survey. Work.

[B12] Patten SB, Svenson LW, Metz LM (2005). Descriptive epidemiology of affective disorders in multiple sclerosis. CNS Spectr.

[B13] Beland Y (2002). Canadian community health survey--methodological overview. Health Rep.

[B14] Canada S (2004). CCHS Cycle 1.1, Public Use Microdata File Documentation.

[B15] Rosati G (2001). The prevalence of multiple sclerosis in the world: an update. Neurol Sci.

[B16] Feeny DH, Torrance GW, Furlong WJ, Spilker B (1996). Health Utilities Index. Quality of Life and Pharmacoeconomics in Clinical Trials.

[B17] Furlong W, Feeny D, Torrance GW, Goldsmith C, Depauw S, Boyle M, Denton M, Zhu Z (1998). Multiplicative Multi-attribute Utility Function for the Health Utilities Index Mark 3 (HUI3) System: A technical Report.

[B18] Horsman J, Furlong W, Feeny D, Torrance G (2003). The Health Utilities Index (HUI(R)): concepts, measurement properties and applications. Health Qual Life Outcomes.

[B19] Feeny D, Furlong W, Torrance GW, Goldsmith CH, Zhu Z, Depauw S, Denton M, Boyle M (2002). Multiattribute and single-attribute utility functions for the health utilities index mark 3 system. Med Care.

[B20] Inc HU (2004). http://www.healthutilities.com.

[B21] Fisk JD, Brown MG, Sketris IS, Metz LM, Murray TJ, Stadnyk KJ (2005). A comparison of health utility measures for the evaluation of multiple sclerosis treatments. J Neurol Neurosurg Psychiatry.

[B22] Maddigan SL, Feeny DH, Johnson JA (2005). Health-related quality of life deficits associated with diabetes and comorbidities in a Canadian National Population Health Survey. Qual Life Res.

[B23] Grootendorst P, Feeny D, Furlong W (2000). Health Utilities Index Mark 3: evidence of construct validity for stroke and arthritis in a population health survey. Med Care.

[B24] Evans RG, Stoddart GL (1990). Producing health, consuming health care. Soc Sci Med.

[B25] Murphy N, Confavreux C, Haas J, Konig N, Roullet E, Sailer M, Swash M, Young C, Merot JL (1998). Quality of life in multiple sclerosis in France, Germany, and the United Kingdom. Cost of Multiple Sclerosis Study Group. J Neurol Neurosurg Psychiatry.

[B26] Rothwell PM, McDowell Z, Wong CK, Dorman PJ (1997). Doctors and patients don't agree: cross sectional study of patients' and doctors' perceptions and assessments of disability in multiple sclerosis. BMJ.

[B27] Patten SB, Beck CA, Williams JV, Barbui C, Metz LM (2003). Major depression in multiple sclerosis: a population-based perspective. Neurology.

[B28] Nortvedt MW, Riise T, Myhr KM, Nyland HI (2000). Performance of the SF-36, SF-12, and RAND-36 summary scales in a multiple sclerosis population. Med Care.

[B29] Svendsen KB, Jensen TS, Overvad K, Hansen HJ, Koch-Henriksen N, Bach FW (2003). Pain in patients with multiple sclerosis: a population-based study. Arch Neurol.

[B30] Ehde DM, Gibbons LE, Chwastiak L, Bombardier CH, Sullivan MD, Kraft GH (2003). Chronic pain in a large community sample of persons with multiple sclerosis. Mult Scler.

[B31] Goldman MD, Cohen JA, Fox RJ, Bethoux FA (2006). Multiple sclerosis: treating symptoms, and other general medical issues. Cleve Clin J Med.

[B32] Kalia LV, O'Connor PW (2005). Severity of chronic pain and its relationship to quality of life in multiple sclerosis. Mult Scler.

[B33] Sprangers MA, de Regt EB, Andries F, van Agt HM, Bijl RV, de Boer JB, Foets M, Hoeymans N, Jacobs AE, Kempen GI, Miedema HS, Tijhuis MA, de Haes HC (2000). Which chronic conditions are associated with better or poorer quality of life?. J Clin Epidemiol.

[B34] Svenson LW, Warren S, Warren KG, Metz LM, Patten SB, Schopflocher DP (2007). Prevalence of multiple sclerosis in First Nations people of Alberta. Can J Neurol Sci.

